# Supplementary material for: Dissecting peri-implantation development using cultured human embryos and embryo-like assembloids
Source: Cell Res. 2023 Jul 17;33(9):661–78. doi: 10.1038/s41422-023-00846-8 (PMC10474050; doi:10.1038/s41422-023-00846-8)
Supplement: Supplementary file 1 — Supplementary information, Fig. S1 [file 41422_2023_846_MOESM1_ESM.pdf]

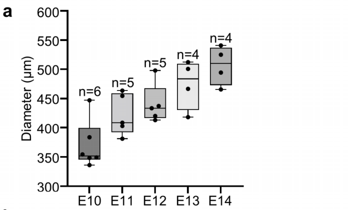

b

Category		TrB	EPI	ExM	XEN
E10	Number (%)	618 (83.0%)	94 (12.6%)	0 (0.0%)	33 (4.4%)
E11		451 (55.0%)	317 (38.7%)	12 (1.5%)	40 (4.9%)
E12		1066 (86.0%)	113 (9.1)	31 (2.5%)	30 (2.4%)
E13		2615 (87.5%)	261 (8.7%)	63 (2.1%)	49 (1.6%)
E14		1490 (52.5%)	707 (24.9%)	623 (22.0%)	17 (0.6%)

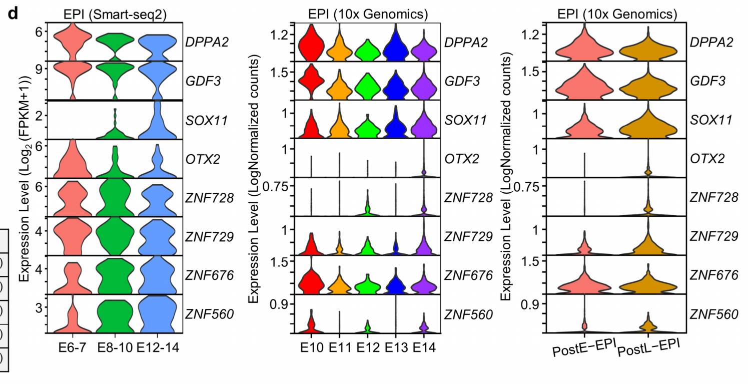

c

Category		PostE-EPI	PostL-EPI	UC	AME1	AME2	PS1	PS2	ExM1	ExM2	VE/YE	AVE
E10	Number (%)	91 (71.7%)	0 (0.0%)	3 (2.4%)	0 (0.0%)	0 (0.0%)	0 (0.0%)	0 (0.0%)	0 (0.0%)	0 (0.0%)	23 (18.1%)	10 (7.9%)
E11		301 (81.6%)	0 (0.0%)	9 (2.4%)	2 (0.5%)	0 (0.0%)	1 (0.3%)	4 (1.1%)	10 (2.7%)	2 (0.5%)	20 (5.4%)	20 (5.4%)
E12		94 (54.0%)	0 (0.0%)	7 (4.0%)	1 (0.6%)	4 (2.3%)	2 (1.1%)	5 (2.9%)	26 (14.9%)	5 (2.9%)	23 (13.2%)	7 (4.0%)
E13		77 (20.6%)	98 (26.3%)	15 (4.0%)	20 (5.4%)	30 (8.0%)	11 (2.9%)	12 (3.2%)	28 (7.5%)	33 (8.8%)	37 (9.9%)	12 (3.2%)
E14		45 (3.3%)	562 (41.7%)	33 (2.4%)	7 (0.5%)	9 (0.7%)	30 (2.2%)	20 (1.5%)	83 (6.2%)	541 (40.2%)	17 (1.3%)	0 (0.0%)

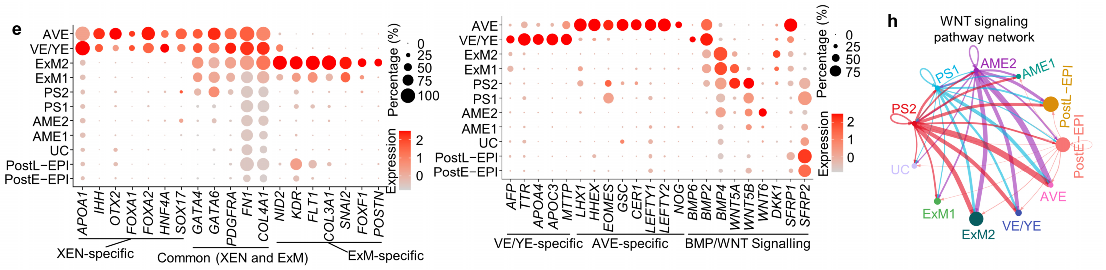

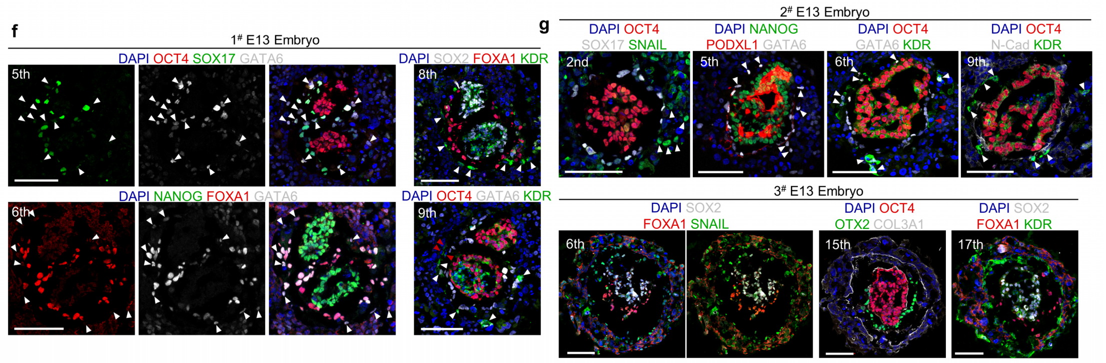

Supplementary information, Fig. S1 Single-cell transcriptomic profiling of 3D-cultured human post-implantation embryos, related to Fig. 1. **a** Diameters of 3D-cultured human embryos used for 10x Genomics sequencing. From embryonic day (E10-14), the number of 3D-cultured human embryos (corresponding thawed blastocysts) collected at each time point was 6 (10), 5 (10), 5 (12), 4 (14) and 4 (17) at each time point, respectively. **b** and **c** Numbers and proportions of different subtypes of cells in extended cultured human embryos at different developmental time points according to the results of scRNA-seq data. **d** Violin plots of indicated genes expressed in EPI by scRNA-seq data. The smart-seq2 results are from published data.[1] **e** Dot plots of candidate genes specific for different cell subtypes. **f** and **g** Immunostaining of EPI, XEN and ExM markers in three E13 extended cultured human embryos. White ordinal numbers indicate section numbers, arrowheads indicate ExM

cells, and red arrowheads indicate few $OCT4^{-}GATA6^{-}KDR^{+}$ ExM cells. **f** a twin embyo; **g** two singleton embryos. We cultured ten E6 blastocysts and obtained three normally developed E13 embryos for immunostaining. **h** The inferred WNT signaling pathway network. Circle sizes are proportional to the number of cells in each subpopulation and line weight represents the communication probability. E, embryonic day; EPI, epiblast; PostE−EPI and PostL−EPI, post-implantation early and late EPI; UC, undefined cell-type; AME, amniotic epithelium; PS, primitive streak; ExM, extraembryonic mesoderm; VE/YE, visceral/yolk sac endoderm; AVE, anterior visceral endoderm; XEN, extraembryonic endoderm. Scale bars, 100 μm.

Reference

1 Xiang, L. *et al.* A developmental landscape of 3D-cultured human pre-gastrulation embryos. *Nature* **577**, 537-542 (2020).
