# Supplementary material for: Dissecting peri-implantation development using cultured human embryos and embryo-like assembloids
Source: Cell Res. 2023 Jul 17;33(9):661–78. doi: 10.1038/s41422-023-00846-8 (PMC10474050; doi:10.1038/s41422-023-00846-8)
Supplement: Supplementary file 2 — Supplementary information, Fig. S2 [file 41422_2023_846_MOESM2_ESM.pdf]

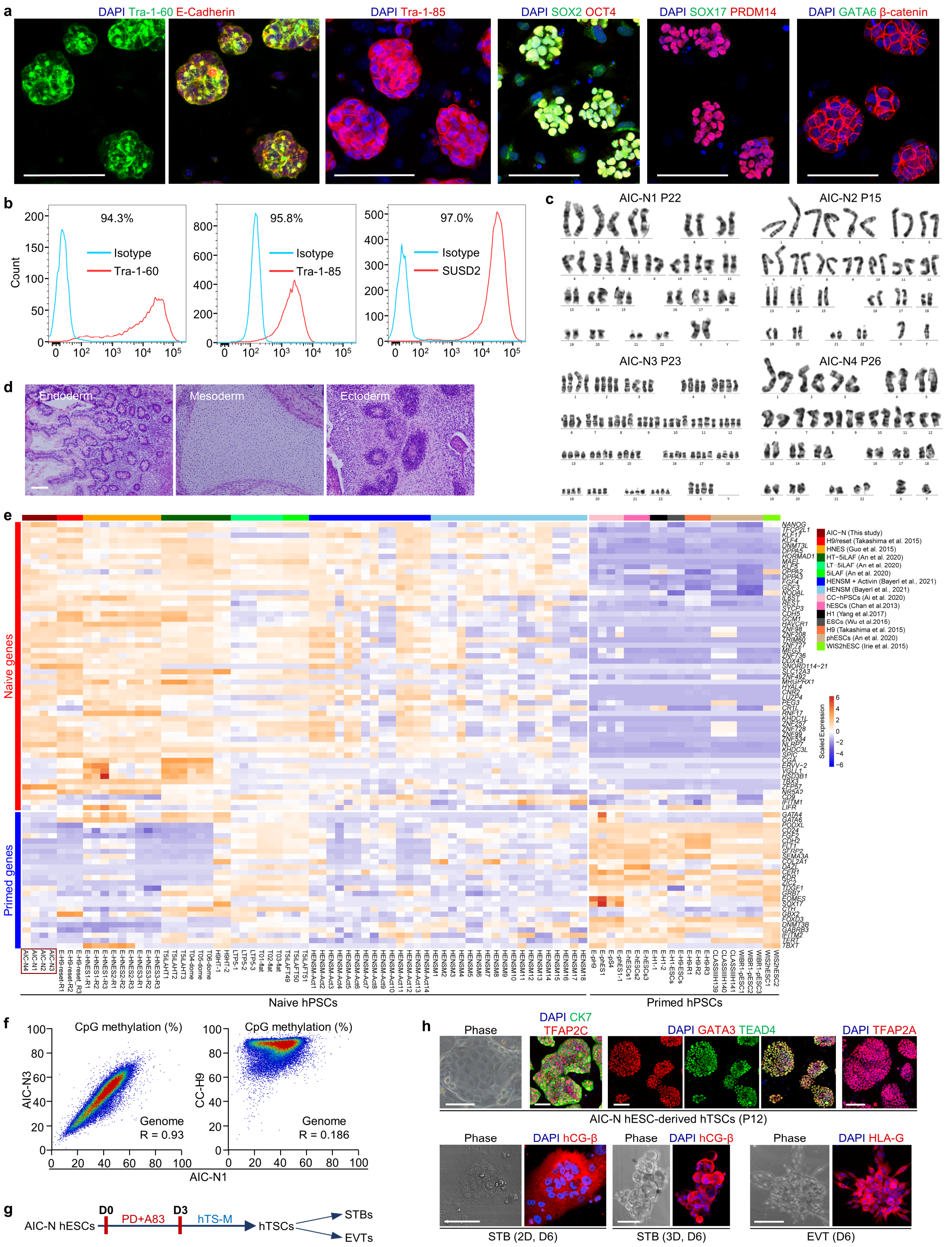

Supplementary information, Fig. S2 Identification of naïve hESCs, related to Fig. 2. a Immunostaining of general pluripotency and hypoblast markers for AIC-N hESCs. **b** Flow cytometry analysis of Tra-1-60, Tra-1-85 and SUSD2 in AIC-N hESCs. **c** G-banding karyotype analysis of four different AIC-N hESC lines. The passage (P) number for karyotyping is indicated. **d** AIC-N hESCs gave rise to teratomas including three germ lineages. **e** Heatmap of representative primed and naïve genes in hPSCs cultured in different conditions. Values represent $\log_2$ (FPKM+1) scaled by gene

expression across samples. **f** Comparisons of global methylation in AIC-N1 (female), AIC-N3 (female) and CC-H9 (female, conventional) hESCs by averaging CpG methylation levels over 50-kb windows. **g** Differentiation schematic of trophoblast lineages from AIC-hESCs. **h** Representative contrast-phase and immunostaining images of different types of trophoblast cells differentiated from AIC-N hESCs. STB, syncytiotrophoblast-like cells; EVT, extravillous cytotrophoblast-like cells. Scale bars, 100 μm.
