# Supplementary material for: Dissecting peri-implantation development using cultured human embryos and embryo-like assembloids
Source: Cell Res. 2023 Jul 17;33(9):661–78. doi: 10.1038/s41422-023-00846-8 (PMC10474050; doi:10.1038/s41422-023-00846-8)
Supplement: Supplementary file 3 — Supplementary information, Fig. S3 [file 41422_2023_846_MOESM3_ESM.pdf]

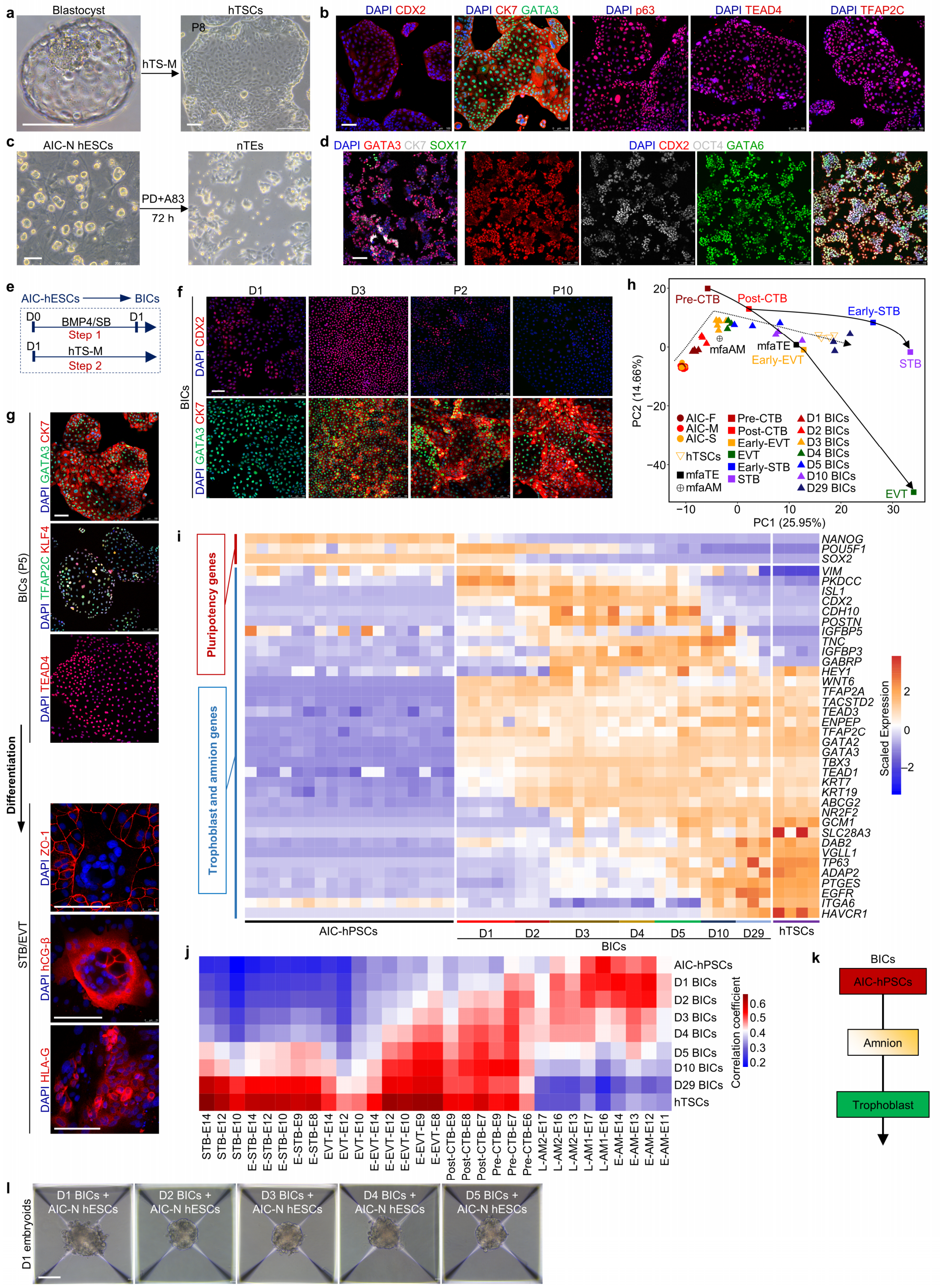

Supplementary information, Fig. S3 Derivation and characterization of extraembryonic cells, related to Fig. 3. a Representative contrast-phase images showing the derivation of human trophoblast stem cells (hTSCs) from a blastocyst.[1] **b** Immunostaining of hTSCs with indicated markers. **c** AIC-N hESCs are induced to differentiate into nTEs (naive hESC-derived trophectoderm-like cells) in PD+A83 for 3 days.[2] **d** Immunostaining of nTEs with indicated markers. **e** Schematic of BIC

(BMP4-induced cell) generation from AIC-hESCs.[3] SB, SB431542; hTS-M, hTSC medium. **f** Immunostaining of BICs with indicated markers at the different time points. **g** Immunostaining of BICs at passage 5 (P5) with indicated markers (top), and differentiation of BICs at P5 into syncytiotrophoblast- and extravillous trophoblast-like cells (bottom). **h** PCA analysis of bulk RNA-seq (AIC-hPSCs[3], hTSCs and BICs different days after induction) and single-cell RNA-seq (trophoblast cells from 3D-cultured human embryos[4], trophectoderm and amnion cells from monkey embryos cultured *in vitro*[5]) data. We filtered the most variable genes among different cells of human embryo with average $\log_2$ (FPKM + 1) > 2 and the square of coeffificient of variation (log CV^2) > 0.2, and 600 most variable genes expressed in both human and monkey embryos were used to perform PCA analysis. **i** Heatmap of representative pluripotency, amnion and trophoblast genes in AIC-hPSCs[3], BICs different days after induction and hTSCs. Values represent $\log_2$ (FPKM+1) scaled by gene expression across samples. AIC-hPSCs contain six cell lines (H9/hES1/S4/h1/h2/h3) cultured in the AIC medium from published data.[3] hTSCs contain two cell lines (hTS1 and hTS2) cultured on feeders or Col IV. BICs are derived from AIC-hESCs cultured on Matrigel (H9/hES1/h1). **j** Heatmap showing Pearson correlation coefficients for expression of variable genes among human trophoblast cells[4], monkey amnion cells[5], AIC-hPSCs, BICs different days after induction and hTSCs. Data of human trophoblast cells and monkey amnion cells are based on average expression level of these single cells. 2035 filtered genes were used as input for Pearson correlation analysis. STB, syncytiotrophoblast; CTB, cytotrophoblast; EVT, extravillous cytotrophoblast; AM, amnion. **k** BICs undergoes a state transition from pluripotency to amnion-like and finally to trophoblast-like fate. **l** Representative contrast-phase images of embryoids assembled from AIC-N hESCs and BICs different days after induction. A large number of dead cells surround the embryoid assembled from D1 BICs and AIC-N hESCs. Scale bars, 100 μm.

Reference

1 Okae, H. *et al.* Derivation of Human Trophoblast Stem Cells. *Cell Stem Cell* **22**, 50-63 e56 (2018).

2 Guo, G. *et al.* Human naive epiblast cells possess unrestricted lineage potential. *Cell Stem Cell* **28**, 1040-1056 e1046 (2021).

3 Ai, Z. *et al.* Modulation of Wnt and Activin/Nodal supports efficient derivation, cloning and suspension expansion of human pluripotent stem cells. *Biomaterials* **249**, 120015 (2020).

4 Xiang, L. *et al.* A developmental landscape of 3D-cultured human pre-gastrulation embryos. *Nature* **577**, 537-542 (2020).

5 Ma, H. *et al.* In vitro culture of cynomolgus monkey embryos beyond early gastrulation. *Science* **366** (2019).
