# Supplementary material for: Dissecting peri-implantation development using cultured human embryos and embryo-like assembloids
Source: Cell Res. 2023 Jul 17;33(9):661–78. doi: 10.1038/s41422-023-00846-8 (PMC10474050; doi:10.1038/s41422-023-00846-8)
Supplement: Supplementary file 4 — Supplementary information, Fig. S4 [file 41422_2023_846_MOESM4_ESM.pdf]

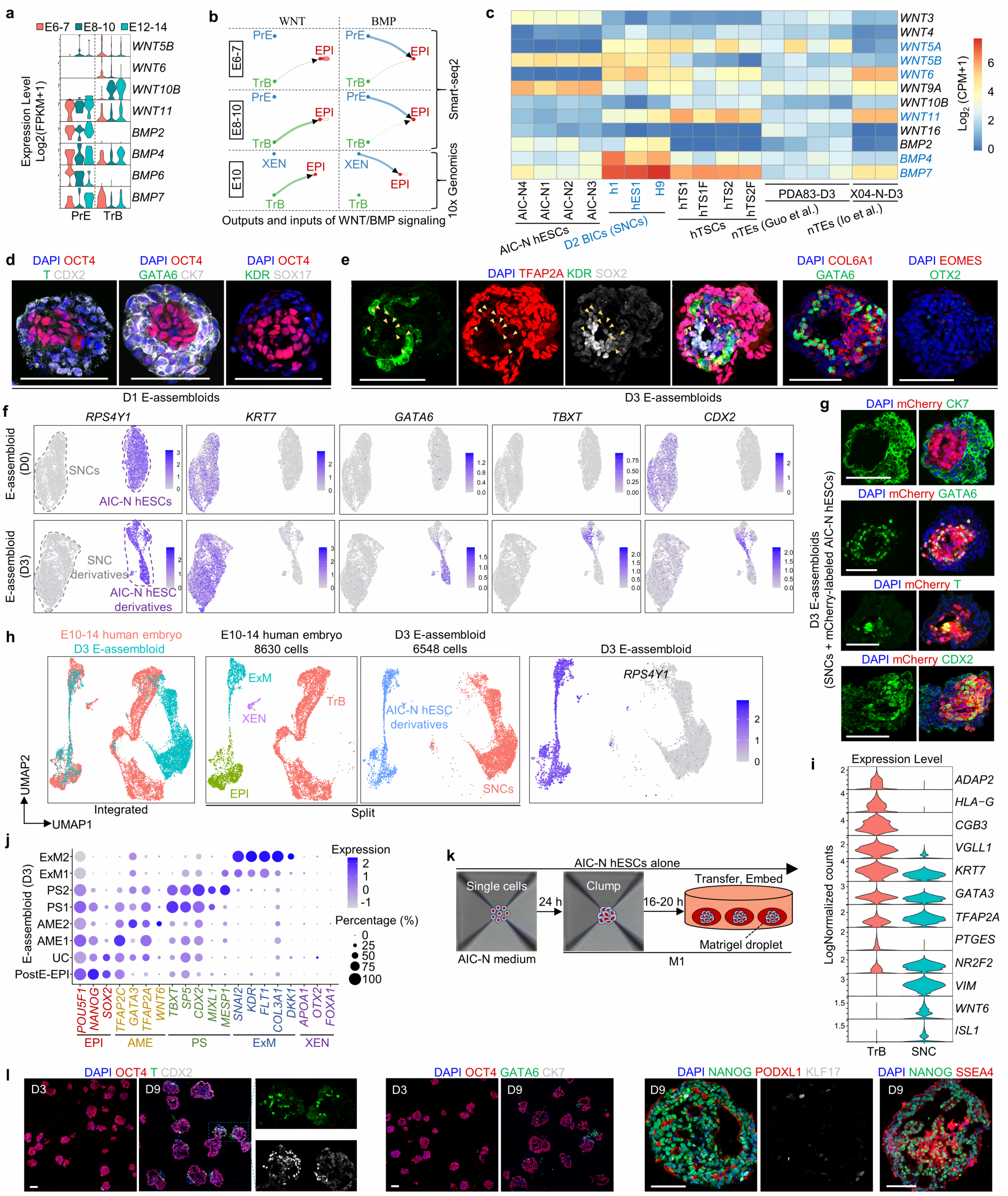

Supplementary information, Fig. S4 SNCs induce the development of AIC-N hESCs in E-assembloids, related to Fig. 3. a Violin plots of WNT and BMP ligand genes in PrE and TrB by scRNA-seq data (smart-seq2).[1] **b** The inferred WNT and BMP signaling outputs and inputs from extraembryonic to embryonic tissues in 3D-cultured human embryos before E10, line weight represents the communication probability. **c** Heatmap of log-transformed expression levels of major ligand genes contributing to WNT and BMP signaling among AIC-N hESCs, and D2 BICs (SNCs), hTSCs cultured on Col IV (hTS1 and hTS2) or feeders (hTS1F and hTS2F), and nTEs (PDA83-D3[2] and X04-N-D3[3]). Values are presented as $\log_2$ (CPM+1). D2 BICs

expressed different levels of *WNT5A/B*, *WNT6*, *WNT11*, *BMP4* and *BMP7*. D2 BICs were used to construct E-assembloids and hereinafter collectively referred to as signaling nest cells (SNCs). **d** and **e** Immunostaining of D1 and D3 E-assembloids cultured in the M1 condition with indicated markers. Yellow arrowheads indicate TFAP2A^{weak}SOX2$^{weak/–}$ AMELCs surrounded by SOX2$^{–}$KDR^{+} ExMLCs. **f** UMAP plots showing expressions of Y-chromosome specific gene *RPS4Y1* and lineage marker genes according to scRNA-seq data from D3 E-assembloids grown in the M1 condition. The E-assembloids were constructed with male AIC-N2 hESCs and female H9 SNCs, and the scRNA-seq data from AIC-N2 hESC clumps and H9 SNCs were used as D0 control. **g** Immunostaining images of D3 E-assembloids grown in the M1 condition showed that CK7 is almost exclusively expressed in mCherry$^{–}$ SNC derivatives, whereas GATA6, T and CDX2 are almost exclusively expressed in mCherry^{+} AIC-N hESC derivatives. E-assembloids are derived from H9 SNCs and mCherry-labeled AIC-N2 hESCs. **h** UMAP visualization and plots of integration analysis of scRNA-seq data from human embryos and D3 E-assembloids. *RPS4Y1* gene labels male AIC-N2 hESC derivatives. **i** Violin plots of indicated genes expressed in TrB (embryo) and SNC (E-assembloid) population by scRNA-seq data (10X Genomics). **j** Dot plots of candidate genes specific for the indicated cell subtypes from D3 E-assembloids. **k** and **l** Diagram (**k**) and representative staining images (**l**) that AIC-N hESC clumps were cultured alone until D3 and D9 in the M1 condition. EPI, epiblast; XEN, extraembryonic endoderm; ExM, extraembryonic mesoderm; TrB, trophoblast; SNC, signaling nest cell; PostE−EPI, post-implantation early EPI; UC, undefined cell-type; AME, amniotic epithelium; PS, primitive streak. Scale bars, 100 μm.

Reference

1 Xiang, L. *et al.* A developmental landscape of 3D-cultured human pre-gastrulation embryos. *Nature* **577**, 537-542 (2020).

2 Guo, G. *et al.* Human naive epiblast cells possess unrestricted lineage potential. *Cell Stem Cell* **28**, 1040-1056 e1046 (2021).

3 Io, S. *et al.* Capturing human trophoblast development with naive pluripotent stem cells in vitro. *Cell Stem Cell* **28**, 1023-1039 e1013 (2021).
