# Supplementary material for: Dissecting peri-implantation development using cultured human embryos and embryo-like assembloids
Source: Cell Res. 2023 Jul 17;33(9):661–78. doi: 10.1038/s41422-023-00846-8 (PMC10474050; doi:10.1038/s41422-023-00846-8)
Supplement: Supplementary file 5 — Supplementary information, Fig. S5 [file 41422_2023_846_MOESM5_ESM.pdf]

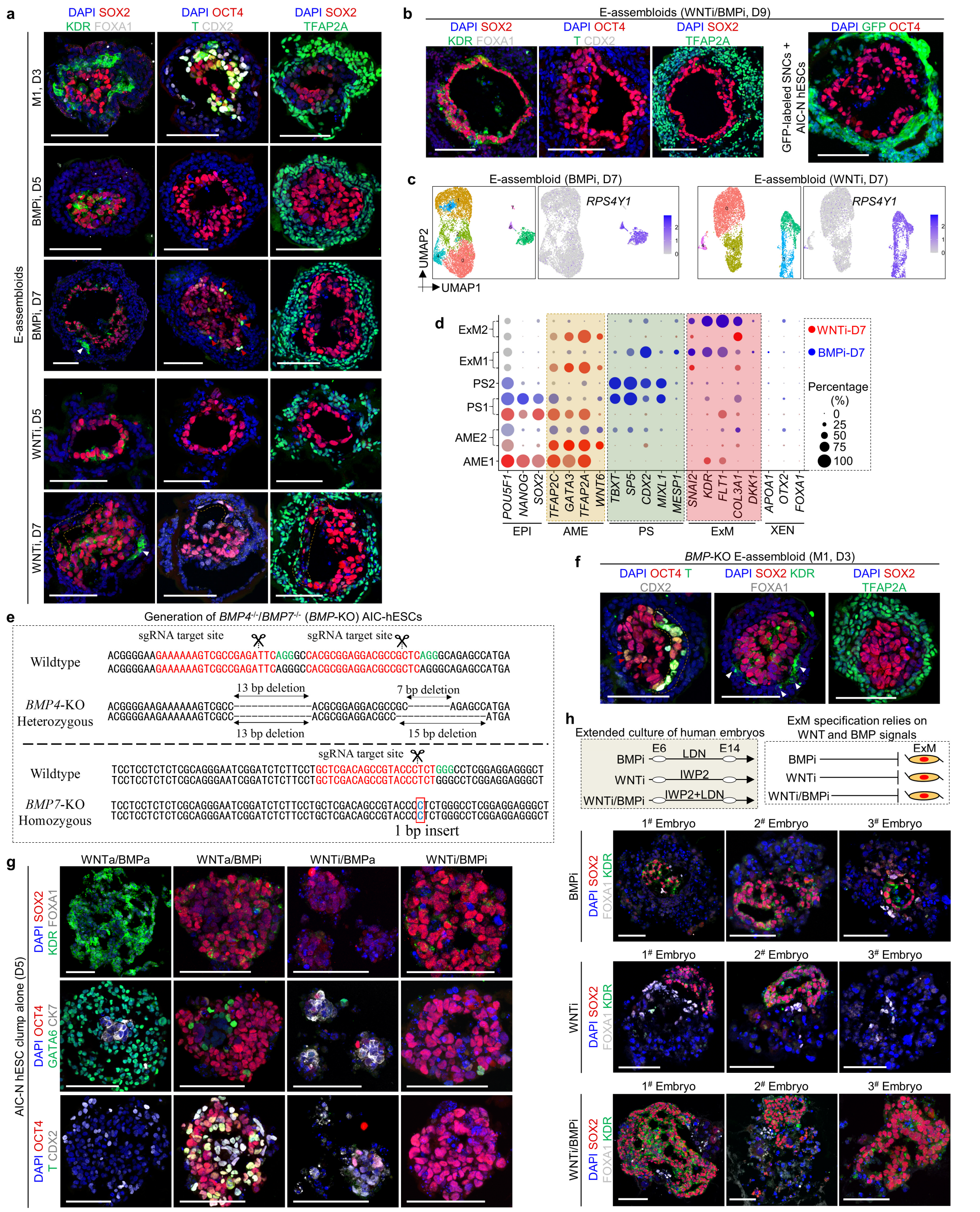

Supplementary information, Fig. S5 Functions of WNT and BMP signaling, related to Fig. 4. a and **b** Representative staining images of E-assembloids grown in indicated conditions at indicated time points. Red and white arrowheads indicate PSLCs and ExMLCs, respectively. Dotted lines show AMELCs. (**b**, right) E-assembloids assembled by AIC-N4 hESCs and GFP-labeled SNCs (from AIC-hES1) showed that almost all AIC-N hESC derivatives expressed the pluripotency marker OCT4 in the WNTi/BMPi condition on D9. **c** UMAP visualization of major subtype of cells and UMAP plots of *RPS4Y1* gene in E-assembloids cultured in indicated

conditions on D7. **d** Dot plots of candidate genes specific for indicated cell subtypes in E-assembloids grown in indicated conditions. **e** Generation of *BMP4*$^{-/-}$/*BMP7*$^{-/-}$ H9 AIC-hESCs (*BMP*-KO H9 AIC-hESCs). **f** Representative staining images of D3 *BMP*-KO E-assembloids grown in the M1 condition. Red and white arrowheads indicate PSLCs and ExMLCs, respectively. Dotted lines show AMELCs. **g** Representative staining images of AIC-N hESC clumps cultured alone in indicated conditions for five days. **h** Schematic diagram of different culture conditions for human embryo (top left); functional diagram of WNT and BMP signaling on ExM specification (top right); and representative staining images of E14 human embryos grown in indicated conditions (bottom). Six blastocysts per group were cultured in the indicated conditions from E6-14, and three surviving embryos per group were used for immunostaining. E, embryonic day; EPI, epiblast; AME, amniotic epithelium; PS, primitive streak; ExM, extraembryonic mesoderm; XEN, extraembryonic endoderm; BMPi, BMP inhibition; BMPa, BMP activation; WNTi, WNT inhibition; WNTa, WNT activation. LDN, LDN193189-2HCl. Scale bars, 100 μm.
