# Supplementary material for: Dissecting peri-implantation development using cultured human embryos and embryo-like assembloids
Source: Cell Res. 2023 Jul 17;33(9):661–78. doi: 10.1038/s41422-023-00846-8 (PMC10474050; doi:10.1038/s41422-023-00846-8)
Supplement: Supplementary file 6 — Supplementary information, Fig. S6 [file 41422_2023_846_MOESM6_ESM.pdf]

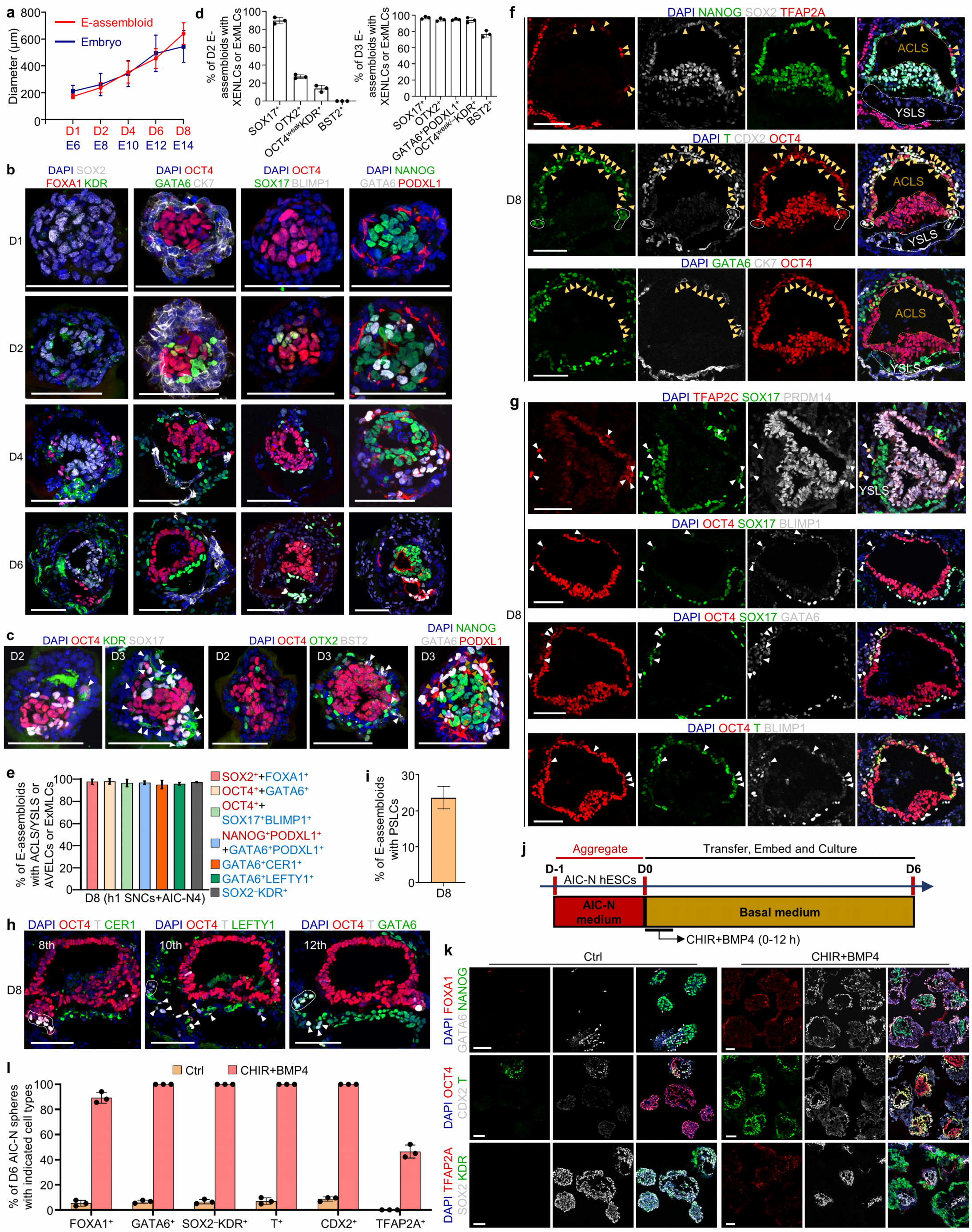


**Supplementary information, Fig. S6 E-assembloids reconstitute 3D structures of early human post-implantation embryos, related to Fig. 6. a** Dynamic diameters of E-assembloids and 3D-cultured human embryos (published data[1]). For E-assembloids, N=3 independent experiments; at least 100 E-assembloids were quantified each experiment; data are presented as mean±s.d. **b** and **c** Representative immunostaining images showing the dynamic development of XENLCs, ExMLCs, ACLS and YSLS in E-assembloids at different time points. **d** Quantification of the E-assembloids with XENLCs or ExMLCs on D2 and D3. Three independent experiments; more than 100

structures were quantified each experiment; data are presented as mean±s.d. **e** Repeated experiments from two other cell lines (h1 SNCs and AIC-N4 hESCs), quantification of the E-assembloids with ACLS and YSLS or AVELCs or ExMLCs on D8. N = 3 independent experiments; more than 100 structures were quantified each experiment; data are presented as mean±s.d. Red and blue represent markers of EPILCs and XENLCs/ExMLCs, respectively. **f** Representative staining images showing the specifications of AMELCs (yellow arrowheads) and PSLCs (white solid lines) in D8 E-assembloids, the yellow and white dotted lines indicate the ACLS and YSLS, respectively. **g** Representative staining images showing the specification of PGCLCs (white arrowheads) in D8 E-assembloids. **h** Representative staining images showing the generation of potential PSLCs (solid lines and arrowheads). White ordinal numbers indicate section numbers. **i** Quantification of D8 E-assembloids with putative PSLCs. N = 3 independent experiments; more than 100 structures were quantified each experiment; data are presented as mean±s.d. **j** Schematic diagram of CHIR+BMP4-treated (12 hours) AIC-N hESC clumps cultured alone for six days. **k** Representative staining images showing the generation of embryonic and extraembryonic lineages in AIC-N hESC-derived spheres. **l** Quantification of the AIC-N hESC-derived spheres with indicated cell types. Three independent experiments; more than 100 spheres were quantified each experimental group; data are presented as mean±s.d. E, embryonic day; AC, amniotic cavity; YS, yolk sac; AVE, anterior visceral endoderm; ExM, extraembryonic mesoderm; PS, primitive streak; LS, -like structure; LCs, -like cells. Scale bars, 100 μm.

## Reference

1 Xiang, L. *et al.* A developmental landscape of 3D-cultured human pre-gastrulation embryos. *Nature* **577**, 537-542 (2020).
