# Supplementary material for: Dissecting peri-implantation development using cultured human embryos and embryo-like assembloids
Source: Cell Res. 2023 Jul 17;33(9):661–78. doi: 10.1038/s41422-023-00846-8 (PMC10474050; doi:10.1038/s41422-023-00846-8)
Supplement: Supplementary file 7 — Supplementary information, Fig. S7 [file 41422_2023_846_MOESM7_ESM.pdf]

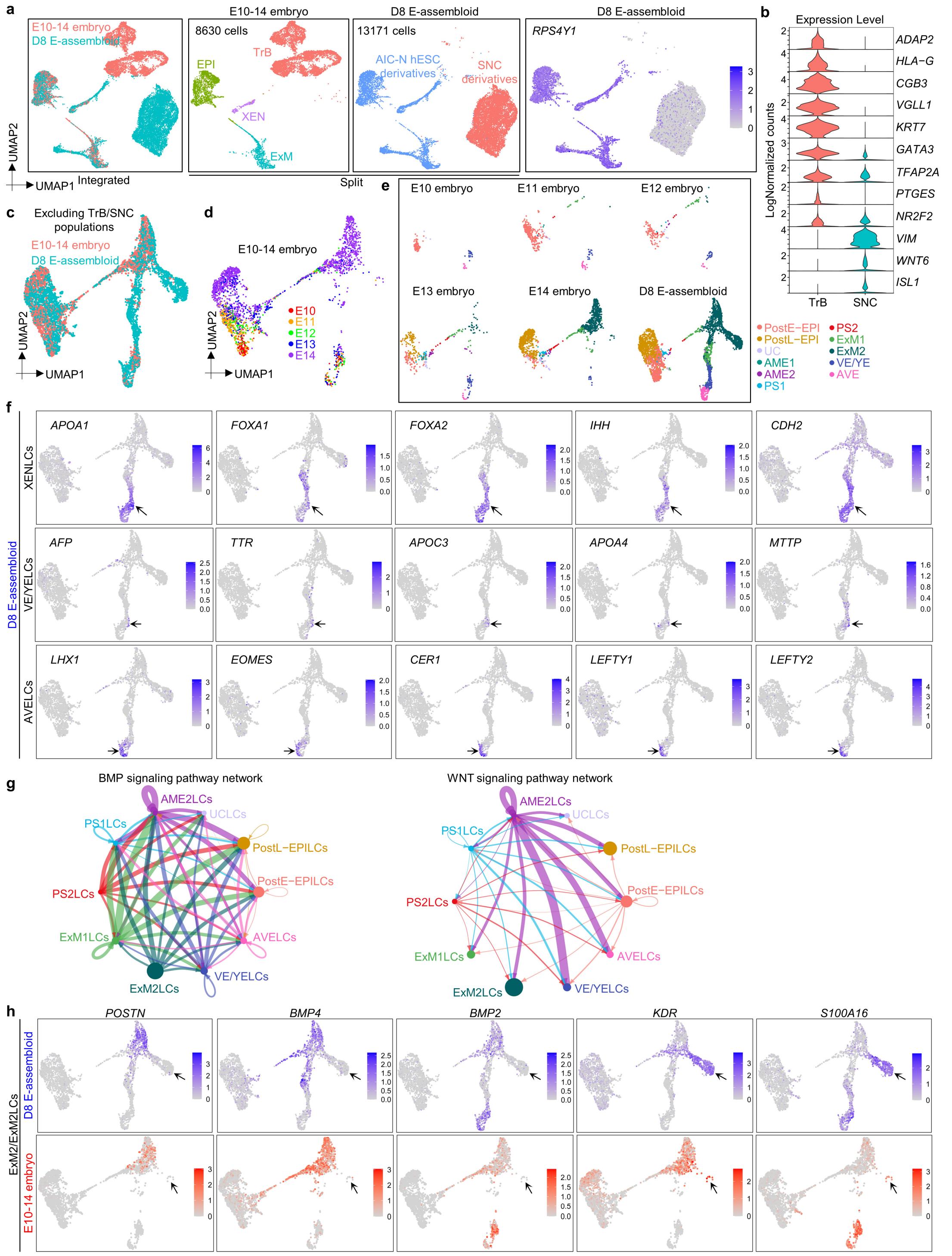


**Supplementary information, Fig. S7 Comparing E-assembloids with 3D-cultured human embryos, related to Fig. 7. a** UMAP visualization and plots of integration analysis of scRNA-seq data from human embryos and D8 E-assembloids. *RPS4Y1* gene labels male AIC-N2 hESC derivatives. **b** Violin plots of indicated genes expressed in TrB (embryo) and SNC (D8 E-assembloid) population by scRNA-seq data (10X Genomics). **c** UMAP visualization of integration analysis of the remaining cell types

after excluding TrB and SNC population in human embryos and D8 E-assembloids, respectively. **d** UMAP visualization of the remaining cell types after excluding TrB population in human embryos at different embryonic days. **e** Split UMAP visualization of the remaining cell types after excluding TrB (E10-14 human embryos) and SNC (D8 E-assembloids) population according to different developmental time points. **f** UMAP plots of indicated genes expressed in D8 E-assembloids. From top to bottom, arrows point to XENLCs, VE/YELCs and AVELCs, respectively. **g** The inferred BMP and WNT signaling pathway networks in D8 E-assembloids. Circle sizes are proportional to the number of cells in each subpopulation and line weight represents the communication probability. **h** UMAP plots of indicated genes expressed in D8 E-assembloids and 3D-cultured embryos. Some cells in ExM2 population exhibit haemato-endothelial characteristics (arrow). EPI, epiblast; XEN, extraembryonic endoderm; ExM, extraembryonic mesoderm; TrB, trophoblast; SNC, signaling nest cell; PostE−EPI and PostL−EPI, post-implantation early and late EPI; UC, undefined cell-type; AME, amniotic epithelium; PS, primitive streak; VE/YE, visceral/yolk sac endoderm; AVE, anterior visceral endoderm; LCs, -like cells.
