# Supplementary material for: Dissecting peri-implantation development using cultured human embryos and embryo-like assembloids
Source: Cell Res. 2023 Jul 17;33(9):661–78. doi: 10.1038/s41422-023-00846-8 (PMC10474050; doi:10.1038/s41422-023-00846-8)
Supplement: Supplementary file 8 — Supplementary information, Table S1 Primer and sgRNA information [file 41422_2023_846_MOESM8_ESM.pdf]

Supplementary information, Table S1: Primer and sgRNA information

**Primer name**	**Primer sequences**
genotyping human *BMP4* Forward	5'-cccagagacaccatgattcc-3'
genotyping human *BMP4* Reversed	5'-cccagagacaccatgattcc-3'
genotyping human *BMP7* Forward	5'-ccctgcgtgtatctctttgtag-3'
genotyping human *BMP7* Reversed	5'-gggaagcctgtttgtagtgaa-3'
**sgRNA name**	**sgRNA sequences**
BMP4 sgRNA1	5'-gaaaaaagtcgccgagattc-3'
BMP4 sgRNA2	5'-cacgcgggaggacgccgctc-3'
BMP7 sgRNA1	5'-gctcgacagccgtaccctct-3'
