# Supplementary material for: Dissecting peri-implantation development using cultured human embryos and embryo-like assembloids
Source: Cell Res. 2023 Jul 17;33(9):661–78. doi: 10.1038/s41422-023-00846-8 (PMC10474050; doi:10.1038/s41422-023-00846-8)
Supplement: Supplementary file 9 — Supplementary information, Table S2 Antibody information [file 41422_2023_846_MOESM9_ESM.pdf]

Supplementary information, Table S2: Antibody information

Antibody (species/isotype)	Source	Cat#	Dilution
TFCP2L1 (goat IgG)	R&D Systems	AF5726	1:500
KLF17 (rabbit IgG)	Atlas Antibodies	HPA024629	1:500
KLF4 (rabbit IgG)	Millipore	09-821	1:600
NANOG (goat IgG)	R&D Systems	AF1997	1:400
SOX2 (rabbit IgG)	Millipore	AB5603	1:600
OCT4 (mouse IgG)	Santa Cruz	sc-5279	1:600
N-Cadherin (rabbit IgG)	GeneTex	GTX127345	1:400
COL3A1 (rabbit IgG)	Abcam	Ab7778	1:400
PRDM14 (rabbit IgG)	Millipore	AB4350	1:200
ALPPL2 (rabbit IgG)[1]	Made in-house (Gao Lab)	N/A	N/A
β-catenin (rabbit IgG)	Cell Signaling Technology	8480	1:200
Tra-1-60 (mouse IgM)	Millipore	SCR001	1:200
E-Cadherin (mouse IgG)	Abcam	ab76055	1:200
BST2 (rabbit IgG)	Abcam	ab243230	1:200
SUSD2-PE (mouse IgG)	Biolegend	327406	1:200
Tra-1-85/CD147 (rabbit IgG)	Abcam	ab108308	1:200
GATA6 (goat IgG)	R&D Systems	AF1700	1:600
GATA6 (rabbit IgG)	Cell Signaling Technology	5851T	1:600
SOX17 (goat IgG)	R&D Systems	AF1924	1:600
SOX17 (rabbit IgG)	Cell Signaling Technology	81778	1:500
GATA4 (goat IgG)	Santa Cruz	sc-1237	1:100
OTX2 (goat IgG)	R&D Systems	AF1979	1:400
FOXA2 (goat IgG)	Santa Cruz	sc-6554	1:200
CDX2 (rabbit IgG)	Abcam	ab76541	1:400
CK7/KRT7 (rabbit IgG)	Abcam	ab181598	1:500
GATA3 (mouse IgG)	Thermo Fisher Scientific	MA1-028	1:500
P63 (mouse IgG)	Abcam	ab735	1:100
TEAD4 (rabbit IgG)	Atlas Antibodies	HPA056896	1:200
TFAP2C (mouse IgG)	Santa Cruz	sc-12762	1:400
HLA-G (mouse IgG)	Abcam	ab52455	1:200
hCG-β (mouse IgG)	Abcam	ab9582	1:200
ZO-1 (mouse IgG)	Thermo Fisher Scientific	33-9100	1:100
Brachyury/T (goat IgG)	R&D Systems	AF2085	1:600
PODXL1 (mouse IgG)	R&D Systems	MAB1658	1:600
EOMES (mouse IgG)	R&D Systems	MAB6166	1:200
TFAP2A (mouse IgG)	Santa Cruz	sc-12726	1:400
LAMININ (rabbit IgG)	Sigma Aldrich	L9393	1:100
SNAIL (goat IgG)	R&D Systems	AF-3639	1:400

VIMENTIN (mouse IgG)	eBioscience	14-9897	1:2000
CER1 (rabbit IgG)	Sigma Aldrich	HPA019917	1:100
LEFTY1 (goat IgG)	R&D Systems	AF746	1:500
BLIMP1 (rabbit IgG)	Cell Signaling Technology	9115S	1:100
SSEA4 (mouse IgG)	Millipore	SCR001	1:100
CD24 (mouse IgG)	Abcam	ab134375	1:100
FOXA1 (mouse IgG)	Abcam	ab55178	1:100
COL6A1 (rabbit IgG)	Abcam	ab182744	1:400
KDR (goat IgG)	R&D Systems	AF357	1:400
Alexa Fluor 488 donkey anti-rabbit IgG	Jackson Immunoresearch	711-545-152	1:600
Alexa Fluor 488 goat anti-mouse IgM	Thermo Fisher Scientific	A-21042	1:600
Alexa Fluor 568 donkey anti-mouse IgG	Thermo Fisher Scientific	A-10037	1:600
Alexa Fluor 488 donkey anti-mouse IgG	Thermo Fisher Scientific	A-21202	1:600
Alexa Fluor 647 donkey anti-rabbit IgG	Thermo Fisher Scientific	A-31573	1:600
Alexa Fluor 555 donkey anti-rabbit IgG	Thermo Fisher Scientific	A-31572	1:600
Alexa Fluor 647 donkey anti-goat IgG	Thermo Fisher Scientific	A-21447	1:600
Alexa Fluor 488 donkey anti-goat IgG	Thermo Fisher Scientific	A-11055	1:600

Reference

1 Bi, Y. *et al.* Identification of ALPPL2 as a Naive Pluripotent State-Specific Surface Protein Essential for Human Naive Pluripotency Regulation. *Cell Rep* **30**, 3917-3931 e3915, doi:10.1016/j.celrep.2020.02.090 (2020).
